# Parasite-mediated interactions within the insect vector: *Trypanosoma rangeli* strategies

**DOI:** 10.1186/1756-3305-5-105

**Published:** 2012-05-30

**Authors:** Eloi S Garcia, Daniele P Castro, Marcela B Figueiredo, Patrícia Azambuja

**Affiliations:** 1 Laboratório de Bioquímica e Fisiologia de Insetos, Instituto Oswaldo Cruz (Fiocruz), Rio de Janeiro, RJ, Brazil; 2 Instituto Nacional de Entomologia Molecular (INCT-EM, CNPq), Rio de Janeiro, RJ, Brazil; 3 Departamento de Química, Instituto de Ciências Exatas, Universidade Federal Rural do Rio de Janeiro, Rio de Janeiro, RJ, Brazil

**Keywords:** *Trypanosoma rangeli*, *Rhodnius prolixus*, Parasite and vector interactions, Parasite development

## Abstract

*Trypanosoma rangeli* is a protozoan that is non-pathogenic for humans and other mammals but causes pathology in the genus *Rhodnius*. *T. rangeli* and *R. prolixus* is an excellent model for studying the parasite-vector interaction, but its cycle in invertebrates remains unclear. The vector becomes infected on ingesting blood containing parasites, which subsequently develop in the gut, hemolymph and salivary glands producing short and large epimastigotes and metacyclic trypomastigotes, which are the infective forms. The importance of the *T. rangeli* cycle is the flagellate penetration into the gut cells and invasion of the salivary glands. The establishment of the parasite depends on the alteration of some vector defense mechanisms. Herein, we present our understanding of *T. rangeli* infection on the vector physiology, including gut and salivary gland invasions, hemolymph reactions and behavior alteration.

## Review

### *Trypanosoma rangeli* biological cycle

The life cycle of *T. rangeli*, which it shares in invertebrate and vertebrate hosts, is complex and mediated by numerous factors, which are still poorly understood [1-4]. Infective parasites have been found mainly in the salivary glands of *R. prolixus*[1-4]; although *T. rangeli* has also been found in the salivary glands of *Triatoma dimiculata* in Colombia [5]. In the invertebrate host, the *T. rangeli* life cycle is characterized in three different regions of the insect vector: the gut, hemolymph and salivary glands, all of which are important for parasite development. The *T. rangeli* interactions in the vector begin with the ingestion of the trypomastigote forms in an infective bloodmeal. The parasites reach the gut of the insect vector and remain in the blood meal for some time after ingestion, but later they transform into epimastigotes that are able to multiply, and then normally cross the intestinal epithelium by an intracellular route and reach the hemocoel [1-4]. Then *T. rangeli* continues multiplying freely in the hemolymph or within hemocytes [6,7], although *T. rangeli* can be destroyed by plasmatocytes [7]. Thereafter, the flagellatesinvade the salivary glands, where they again multiply, and finally transform into metacyclic trypomastigotes, the forms that can be transmitted to mammalian hosts during a blood meal through salivary secretion [1-4,8-10].

## Hemocoel invasion

**Figure 1:**
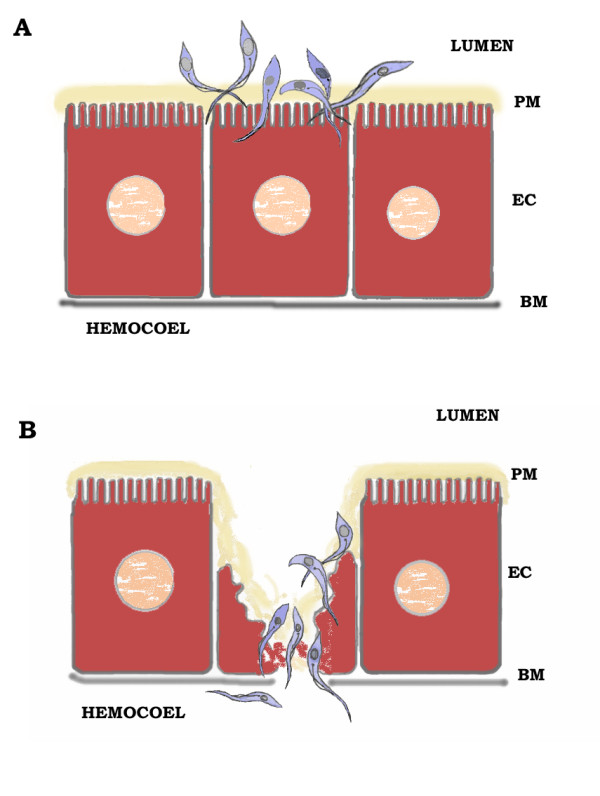
**Illustration of*Trypanosoma rangeli*adhesion (A) and invasion (B) to*Rhodnius prolixus*gut epithelial cells.***T. rangeli* epimastigote forms bind to the epithelium cells (EC) through recognition of lectins from the extracellular membrane layers (perimicrovillar membranes – PM), for subsequent attachment and invasion. BM – basal membrane.

In order to complete its developmental cycle in the insect vector*, Trypanosoma rangeli* needs to invade the hemocoel to overcome gut defense reactions [11-13]. Although it is known that *T. rangeli* decreases the growth of *R. prolixus* microbiota [6,13], the mechanism that facilitates the survival and passage of the parasite from the lumen of the gut to the hemolymph needs further investigation. The midgut epithelium and possibly the gut perimicrovillar membrane of the vector gut represent fundamental steps in the life cycle of *T. rangeli* since they are related to the passage of the parasite from the midgut lumen to the hemocoel (Figure 1). Gamma irradiation causes changes in the ultrastructural organization of perimicrovillar membranes and microvilli in the gut, which leads to earlier parasite infection in the hemolymph in irradiated insects [14]. The ability of *T. rangeli* to attach to the gut surfaces of the insect vector is important for its development. Before invading epithelial tissues and/or cells epimastigotes have to find ways to attach to the gut lumen. Experiments using scanning electron microscopy have demonstrated that *T. rangeli* attaches to the surface of some epithelial cells recognized by the parasites, for subsequent attachment and invasion. The parasites bind to the epithelium through the extracellular membrane layers (perimicrovillar membranes), and not to the plasma membrane layers [14,15] (Figure 1). The presence of certain cells in the gut epithelium of *R. prolixus* is somehow recognized by the parasites for subsequent attachment and invasion [14,15]. In addition, some damage in the intestinal epithelium appears after parasite attachment [6,15] (Figure 1). However, other data have demonstrated that the penetration of *T. rangeli* into the gut depends on traversing the epithelial cells by an intracellular route without damaging the cells [8]. But *in vivo* and *in vitro* experiments have shown an association of several parasites with the same gut cell, and close contact between the parasites and the membrane layers. Also on cell penetration *T. rangeli* damages the surface, moving within the cytoplasm of the epithelial cell, and always in direct contact with the cytoplasmic organelles rather than the endocytic vacuoles (Figure 1). When the parasites reach the basal region, they cross the basal lamina, and enter the hemocoel [6,8,15] (Figure 1). The success of *T. rangeli* to invade the insect hemocoel depends on both the parasite strain and the triatomine species. Usually, the insects are more susceptible to strains isolated from the same geographical region [16]. Even so, the invasion rate of the gut into the hemocoel is low, only about 10% of parasites pass through the gut cell wall. The remaining parasites present in the gut lumen are excreted with the digested blood meal [1-4,8].

## Hemolymph interactions

Although *R. prolixus* has an efficient system to eliminate pathogenic microorganisms, *T. rangeli* has the ability to survive in the hemolymph of *R. prolixus* counteracting the defense responses in many ways, and reaching the salivary glands to complete its life cycle in the invertebrate host [1-4,6,17-19]. *In vivo* and *in vitro* experiments have shown that oral infection with *T. rangeli* followed by inoculation of the insects with the same parasite inhibits hemocyte microaggregation reactions and release of arachidonic acid into the hemolymph of *R. prolixus*[18,19]. Additionally, a *T. rangeli* oral infection significantly reduces the phagocytic activities of *R. prolixus* hemocytes by inhibition of the PAF and eicosanoids pathways [20]. The mechanisms of this inhibition process is unknown, but some studies suggest that nitric oxide and superoxide could be the signaling molecules responsible to take the message from the gut to the hemocoel regulating anti-parasite reactions in this region [21,22].

**Figure 2:**
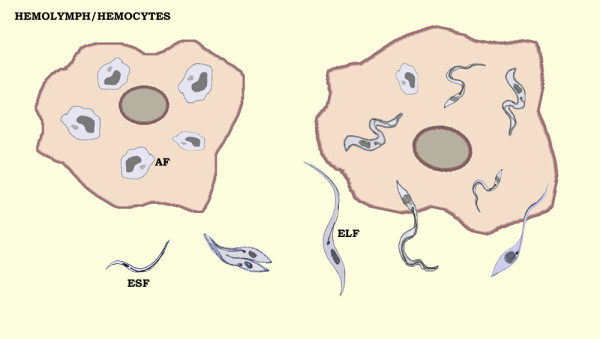
**Illustration of*Trypanosoma rangeli*inside hemocytes and in the hemolymph of*Rhodnius prolixus*hemocoel.** The parasite survives and multiplies freely in the hemolymph or penetrates into the hemocytes, especially plasmatocytes. *T. rangeli* epimastigote short forms (ESF) transform into amastigote forms (AF) or epimastigote long forms (ELF) which are able to invade the salivary gland.

Once in the hemocoel, the parasite survives and multiplies freely on the hemolymph or penetrates into the hemocytes, especially plasmatocytes [6,7] (Figure 2). *T. rangeli* has been shown to overcome the hemolymph reactions, avoiding responses like, lysozyme and trypanolytic activity [23-26], prophenoloxidase (proPO) activation [18,25-27], phagocytosis and hemocyte microaggregation [18,19,28], hemolymph agglutination [23,29,30] and superoxide/nitric oxide generation [21,22]. All these immune reactions are reduced by the parasite infection.

Some studies have revealed the presence of the short epimastigote forms of *T. rangeli* in the hemolymph during the first hours after hemocoel infection (Figure 2). After this period they transform into long epimastigote forms that are able to invade the hemocytes as well as the salivary glands [25,26] (Figure 2). The inoculation of the short epimastigote forms was able to activate the *R. prolixus* proPO system in the hemolymph while the long form was not [26]. This can be explained in part by the presence of a galactose-binding lectin purified from *R. prolixus* hemolymph that affects the survival and motility of short but not long *T. rangeli* epimastigotes forms [31].

*Rhodnius* species and/or *T. rangeli* strains may both be considered key factors for completing the parasite development in the vector [32,33]. Recent investigations have demonstrated that the DNA mini satellites of the parasite may be involved in these interactions. The incubation of *T. rangeli* strains with *R. prolixus* hemolymph indicates the presence of a trypanolytic activity which acts against a *T. rangeli* KP1- isolated from *R. colombiensis*, but has no lytic activity against the *T. rangeli* KP1+ strain from *R. prolixus,* both species were from Colombia. The survival of the latter strain suggests that hemolymph of *R. prolixus* seems to be a biological barrier which does not allow the development and transmission of KP1- [32,33].

## Invasion of salivary glands

**Figure 3:**
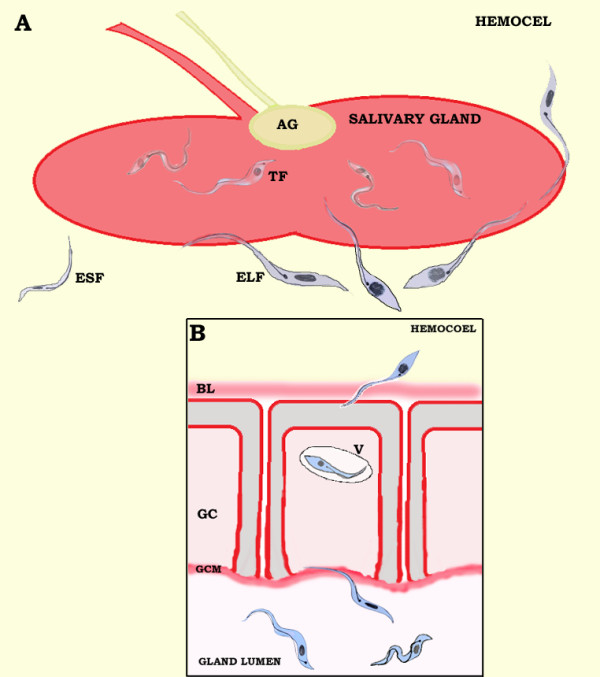
**Illustration of*Trypanosoma rangeli*adhesion (A) and invasion (B) into the*Rhodnius prolixus*salivary gland.** To cross the basal lamina (BL) that surrounds the salivary glands the parasite penetrates via the flagellum and invaginates in a vacuole (V) in which the trypanosome crosses the gland cells (GC) to reach the central lumen. After reaching the gland lumen, the parasite epimastigote long forms (ELF) remain adhered to the gland cell microvilli (GCM) by their flagella, while metacyclic trypomastigote forms (TF) are found swimming freely in the saliva. AG – accessory gland; ESF – epimastigote short forms.

Detailed cytochemical characterization of *Triatoma infestans* and *Panstrongylus megistus* salivary gland cells have been described [34]. Invasion into the insect vector salivary glands by *T. rangeli* is necessary to complete its cycle and transmission to mammals, which is mediated by specific receptor-ligand interactions [35]. *T. rangeli* penetrates the *R. prolixus* salivary glands via the outer "membranes" disrupting the inner layers, in order to cross the basal lamina that surrounds the salivary glands and invades through the gland cells cytoplasm [35,36] (Figure 3). The parasite penetrates the flagellum foremost and then invaginates the gland cell to create a vacuole in which the trypanosome crosses the gland cells to reach the central lumen (Figure 3). After reaching the gland lumen, the epimastigotes remain adhered to the gland cell microvilli by their flagella, while metacyclic trypomastigotes are found swimming free in the saliva [35,36] (Figure 3). Epimastigotes cross the basal lamina through small holes to reach the glandular epithelium, which suggests that they produce a lytic molecule to allow them to pass through the epithelial barrier [35-37].

*R. prolixus* salivary glands are highly glycosylated and most protozoans have glycosylated compounds, as lectins or lectin-like molecules and enzymes, to regulate the parasite adhesion or invasion to host cells [38]. Since epimastigoteforms invade salivary glands, ecto-phosphatase activity of long epimastigote forms could be involved at the interaction sites of parasites and salivary glands with d-galactose and specific lectin-receptors [39]. Knowledge about salivary gland structures facilitates studies on the role of surface molecules in the attachment/invasion process by *T. rangeli*[38]. *Rhodnius* salivary glands have been shown to be rich in carbohydrate moieties on their surface, and present diverse lectin binding patterns with specific carbohydrate residues in the basal, muscle, and cell layers of the glands [38,40]. The carbohydrates detected on the salivary gland surface were used to investigate the adhesion between *T. rangeli* and the *R. prolixus* salivary glands. Experiments *in vitro* on attachment inhibition assays using long epimastigotes (the invasion/adhesion forms) demonstrated that some carbohydrates used were capable of inhibiting the receptors on both the salivary glands and *T. rangeli* surfaces [40]. *R. prolixus* salivary glands have several lectins which present surface-related sugars, and diverse carbohydrate residues are present in the basal lamina, muscle, and cell layers of the gland. Incubation of Con A reacted intensely with the whole salivary glands and with basal membrane in particular, indicating high concentrations of mannosyl residues [38,40]. *In vitro* sugar inhibition assays have demonstrated that some sugars tested attached to the surface of *R. prolixus* salivary glands and/or *T. rangeli* and inhibited the adhesion of the long epimastigotes to the gland surface [40]. The attachment inhibition tests, using parasites or salivary glands pre-treated with sugar revealed that the highest inhibitory activities were observed with N-acetyl-D-glucosamine, N-acetyl-D-galactosamine, and galactose. These molecules may serve as receptors by which long forms of *T. rangeli* epimastigotes attach to the salivary gland surface, prior to invasion [38,40].

## Saliva and alterations in behavior

To overcome vertebrate reactions that prevent blood loss, saliva of *R. prolixus* contains dozens of different compounds with antihemostatic action, such as anticoagulants, antiplatelet and anti-inflammatory activities, and vasodilator compounds. Many of these biologically active salivary proteins belong to the lipocalin protein family [41-43]. The ultimate effect of this salivary antihemostatic apparatus is faster feeding by *R. prolixus* by decreasing the time required by the insect to locate the skin blood vessels and sucking blood efficiently [9,44]. The efficiency of salivary parasite transmission is increased by prolonged intradermal probing time on the host by infected insect vectors. *R. prolixus* infected with *T. rangeli* displays enhanced probing time, and that infection of the salivary glands affected the feeding behavior of the vectors increasing the number of intradermal piercings on a rabbit host reducing the ability to suck the blood when the insect is infected with the flagellate [9,45]. The prolongation of the probing time and the reduction of blood ingested in infected insects were not correlated to either the health of the insects or a physical obstruction of the food channel by the parasites by demonstrating that infected bugs probed and fed normally on a membrane artificial feeder [9]. Thus, it is probable that a *T. rangeli* infection causes salivary gland pathology that must contribute to transmission efficiency. In fact, saliva production was drastically reduced in insects with salivary glands infected with *T. rangeli*, as evaluated by plasma clotting time, apyrase activity, and NO-like compounds [9,41-43]. Since *T. rangeli* may damage the salivary gland cells of *R. prolixus*[8], salivary gland lesions could cause a deficiency in the biosynthesis processes of saliva components [9]. Thus, *T. rangeli* impairs *R. prolixus* salivary gland function, preventing full expression of its antihemostatic machinery. This led the insects to prolong the duration of intradermal probing, which favors *T. rangeli* transmission. Finally, *T. rangeli* infection in the hemolymph of *R. prolixus* leads to a delay in molting, alters insect movements and can increase mortality [46-48].

## Future perspectives

There is ongoing research in our laboratory on the direct and indirect interactions between *T. rangeli* and *R. prolixus* in the insect gut, hemolymph and salivary glands, especially on the parasites cell tissue invasion. The flagellate mechanisms of the inhibition of cellular and humoral immune reactions in this insect vector need further investigation. However, it is becoming clear that some aspects of these gut and salivary gland invasions as well as the hemolymphatic compounds involved in this interaction are known. However, ligand-molecule interactions and several other surface molecules required for invasion of the gut and salivary glands must be better characterized. Additionally, epithelial immune reactions against the parasite invasion are poorly understood. Now, the increased availability of *Rhodnius* and *T. rangeli* functional genome analyses in combination with new experimental models, including double-stranded RNA knockdown, RNAi screens, transcriptomic and proteomic approaches, transgenesis, paratransgenesis of the vector and/or parasite, will offer a powerful tool for elucidating these parasite – vector interactions. Understanding how parasites are recognized as non-self, and how they have developed in the vector and the transmission strategies, facilitates the description of the molecular parasite-vector interface, vector competency, and provides unique opportunities to investigate the role of *T. rangeli* in shaping *R. prolixus* reactions against the parasite.

## Competing interest

The authors declare that they have no competing interests.

## Authors’ contributions

ESG, DCP, MBF, and PA conceived the review design, wrote the drafts and approved the final manuscript.
